# Visual impairment from fibrous dysplasia in a middle-aged African man: a case report

**DOI:** 10.1186/1752-1947-3-14

**Published:** 2009-01-13

**Authors:** Charles O Bekibele, Olubayo A Fasola, Vickie N Okojie, Opeyemi O Komolafe, Olayiwola A Oluwasola, Jude K Emejulu, Ayotunde I Ajaiyeoba, Aderonke M Baiyeroju

**Affiliations:** 1 Department of Ophthalmology, University College Hospital, Ibadan, Nigeria; 2 Maxillofacial Surgery, University College Hospital, Ibadan, Nigeria; 3 Morbid Anatomy, University College Hospital, Ibadan, Nigeria; 4 Neurosurgery, University College Hospital, Ibadan, Nigeria

## Abstract

**Introduction:**

Fibrous dysplasia is a benign tumour of the bones and is a disease of unknown aetiology. This report discusses a case of proptosis and visual deterioration with associated bony mass involving the right orbit.

**Case presentation:**

A 32-year-old Nigerian man of Yoruba ethnic origin presented to the eye clinic of our hospital with right-eye proptosis and visual deterioration of 7-year duration. Presentation was preceded by a history of trauma. Proptosis was preceded by trauma but was non-pulsatile with no thrill or bruit but was associated with bony orbital mass. The patient reported no weight loss. Examination of his right eye showed visual acuity of 6/60 with relative afferent pupillary defect. Fundal examination revealed optic atrophy. Computed tomography showed an expansile bony mass involving all the walls of the orbit. The bony orbital mass was diagnosed histologically as fibrous dysplasia. Treatment included orbital exploration and orbital shaping to create room for the globe and relieve pressure on the optic nerve.

**Conclusion:**

Fibrous dysplasia should be considered in the differential diagnosis of slowly developing proptosis with associated visual loss in young adults.

## Introduction

Fibrous dysplasia is a benign, slowly growing disorder of bone in which the normal cancellous bone is replaced by immature woven bone and fibrous tissue [1]. This condition was first reliably recognized by von Recklinghausen in 1891 [2]. Since then, a large number of cases have been reported and considerable advances have been made in the understanding and treatment of the disease [3] which constitutes 2.5% of all bone tumour and 7.5% of all benign bone neoplasms [4]. It has no sex preference [3] and usually manifests before the 3rd decade of life [5]. Fibrous dysplasia has two basic clinical forms, namely the monostotic and the polyostotic forms [3]. The monostotic form of this disease constitutes about 70% of cases and only involves the craniofacial skeleton in about 10% of cases, having a predilection for the ribs and femur [3].

Histological examination provides the basis for an accurate diagnosis. The tumour is characterized by multiple small and irregular spicules of immature bone superimposed on a background of moderately cellular fibrous connective tissue [6]. However, ancillary investigations, like computerized tomography (CT) which shows the characteristic 'ground glass' appearance in the sclerotic form and non-homogenous appearance in the cystic and mixed form, may be needed to complement findings of histopathology.

Fibrous dysplasia may cause ophthalmic problems such as proptosis and dystopia, ocular motility problem and cosmetic deformity; however, visual loss represents the most common neurological complication of fibrous dysplasia affecting the skull [7].

Fibrous dysplasia, though not rare, is a disease mainly documented among Caucasians [1,3,8] and Asians [9]; few reports are found in the literature of this problem among African Nigerians [10]; especially of the monostoic form with primary orbital involvement.

## Case presentation

A 32-year-old Nigerian businessman of Yoruba ethnic origin was referred to the eye clinic of our hospital from another hospital in Nigeria with complaints of progressive protrusion of the right globe for 7 years. Six months prior to the onset of his complaints, he had hurt the edge of the right superior orbital margin against the edge of an iron bed at boarding school. No treatment was received for this. The protrusion of the globe continued to increase for about 7 months and then stopped. There was no pain and no diplopia but there had been progressive deterioration of the vision in the eye. Initial exploration of the right orbit performed at the referring hospital revealed a bony hard mass involving both the lateral and medial orbital wall. This mass could not be removed. There was no history of weight loss, heat intolerance or excessive weight gain. The proptosis was not made worse by the Valsalva manoeuvre. The patient experienced no unusual noises in the head. He had no history of swelling (of bony or soft tissue) in other parts of the body. Furthermore, there were no hoarseness of voice, dysphagia, cough, palpitation, headache, vomiting or seizure and no focal neurological deficits. The patient had no known hypersensitivities and no diabetes or asthma. He was single, the first of 6 children of his parents. His father had died at an age of about 62 years of an unknown cause, the mother was alive and well, aged about 60 years. There was no family history of similar eye problems.

**Figure 1:**
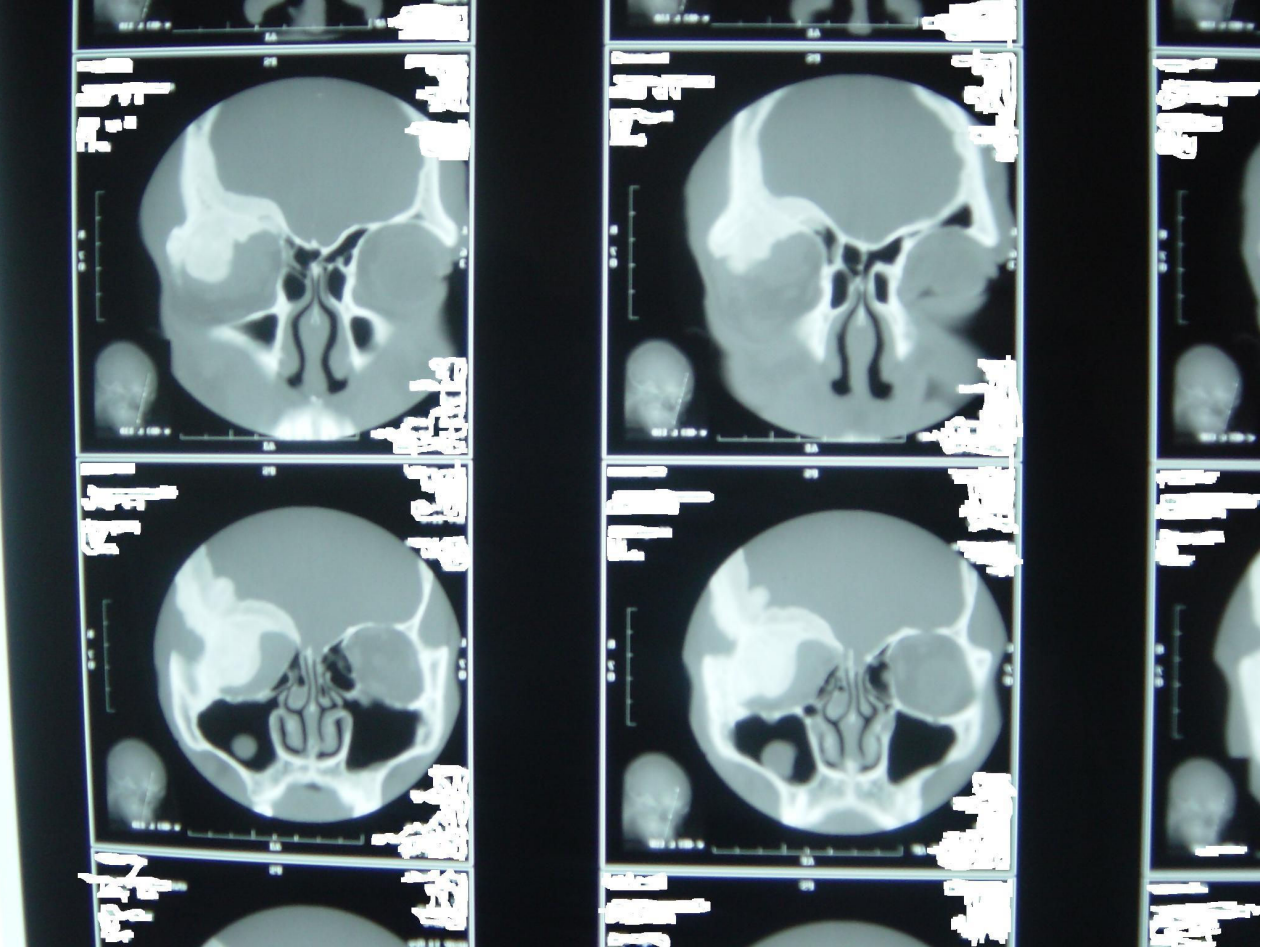
**Preoperative cranial computed tomography scan showing expansile bony mass of the right orbit involving the greater wing of the sphenoid, the medial wall of the orbit and the greater wing of the sphenoid (frontal view)**. There is encroachment into the retro-orbital space with obvious proptosis of the right globe. There is no evidence of intracranial extension.

**Figure 2:**
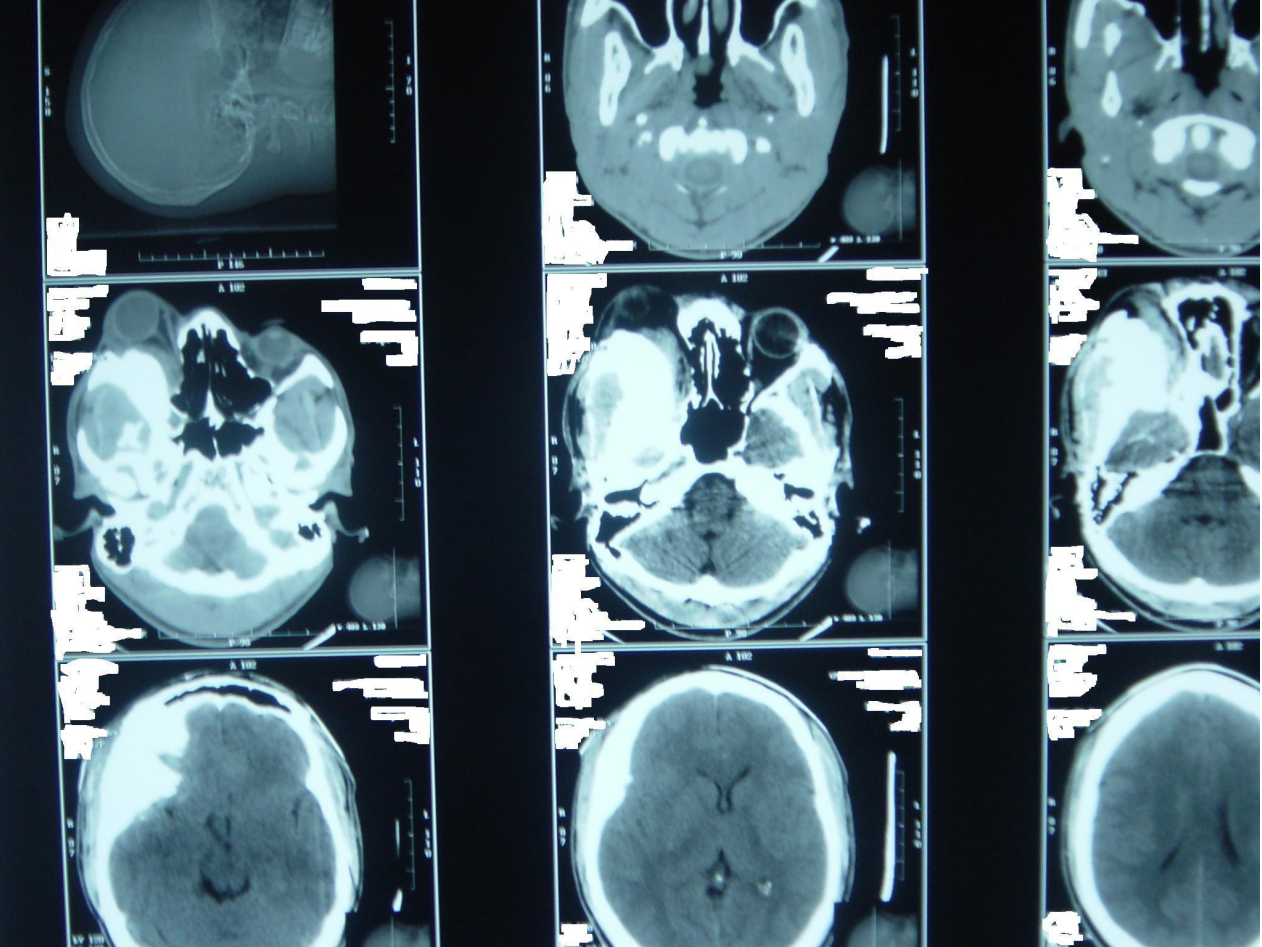
**Preoperative cranial computed tomography scan showing expansile bony mass of the right orbit involving the greater wing of the sphenoid, the medial wall of the orbit and the greater wing of the sphenoid (transverse view)**. There is encroachment into the retro-orbital space with obvious proptosis of the right globe. There is no evidence of intracranial extension.

Examination revealed an otherwise healthy-looking man, with normal systemic examination. The right ocular examination revealed a visual acuity of 6/60 with a proptosis of 17 mm (Hertel exophthalmometer). The proptosis was non-axial (inferotemporally), non-retropulsive, nonpulsatile, nontender and had no thrill and no bruit. There was chemosis of the overlying conjunctiva with moderate restriction of the extra-ocular muscle movement in all direction of the gaze. The pupillary reaction was sluggish with a relative efferent pupillary defect. Fundoscopy showed a pale disc with distinct margins. The left eye had a visual acuity of 6/5 with normal anterior and posterior segments. Cranial computed tomography scan (Figures 1 and 2) showed a right expansile bony mass involving 1) the orbital roof and especially the lesser wing of the sphenoid; and 2) the medial and lateral walls of the orbit, especially the greater wing of the sphenoid. There was partial inferior encroachment involving the right retro-orbital space with compression of the globe against the medial orbital wall. No intracranial extension was seen. A clinical diagnosis of fibrous dysplasia was made.

**Figure 3:**
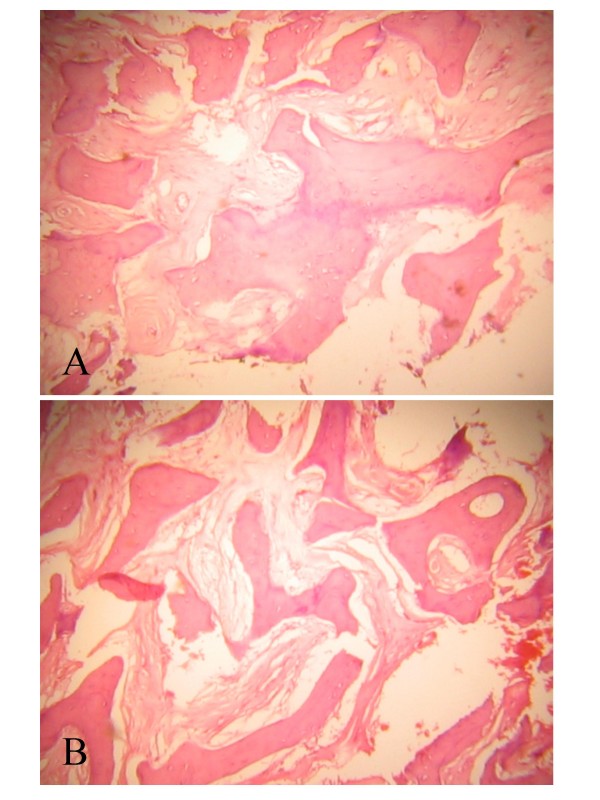
**Histology photograph of specimen obtained at surgery, showing broad sheets of trabeculae of calcified bone with sparsely cellular intervening connective tissue stroma**.

The ophthalmic, neurosurgical and maxillofacial units of our hospital carried out a joint surgical exploration of the right orbit through a modified lateral orbitotomy, using an electric drill for the lateral orbital wall. Operative findings included thickened expanded zygomatic bone and greater wing of sphenoid, with the orbit being almost completely obliterated with expanded dense bony tissue. Gradual removal of the bony mass was performed in layers, using a hammer and chisel until a new orbital space was created. The histological finding (Figure 3) was in keeping with fibrous dysplasia and consisted of broad sheets of interconnection trabeculae of calcified bone with sparsely cellular intervening vascular connective tissue stroma. Postoperatively, there was reduction in the degree of proptosis. The patient was discharged after 21 postoperative days at which time the proptosis had reduced to 6 mm (Hertel exophthalmometer). At the time of writing, his postoperative visual acuity remains at 6/60 and he is receiving follow-up in the outpatient clinic.

## Discussion

Fibrous dysplasia results from a defect in osteoblastic differentiation affecting the final maturation of the bone [11]. Although described as a non-familiar, congenital disorder of the bone, it usually manifests before the 3rd decade of life [6]. Our case fell within the age group described in the literature. The history of trauma preceding the onset of the pathology in this case may be of interest. This is because there had also been a few reports describing a cause-and-effect association between fibrous dysplasia and trauma [12]. However, the 'bumping into objects' described by the patient may be due a pre-existing visual impairment or field defect in the affected eye which was not noticed by the patient until the incident of trauma; even more so as there was no objective visual acuity or field assessment prior to the period. Trauma during puberty when bone development is at its maximum may have implications on the development of tumours of the bone, but this may be difficult to establish in this case as bony growth should have concluded prior to the age when he sustained the trauma.

The rapid worsening of visual acuity as described in this case could be as a result of cyst formation within the tumour, with resultant compression of the optic nerve and impairment of the venous return from the orbit. This is supported by the fact that there was lot of conjuctival chemosis which resolved after surgical decompression of the orbit. The loss of visual acuity may also have been a result of haemorrhage into the tumour resulting from the trauma sustained. However, this could not be substantiated from the histology results. Visual impairment following fibrous dysplasia has been attributed to many factors which include optic-nerve traction due to proptosis, sinus mucocele formation with raised intra-orbital pressure, haemorrhage within the tumour, optic canal stenosis, as well as cyst formation within the lesion [13].

Establishing the diagnosis of fibrous dysplasia requires close cooperation between clinician, radiologist and pathologist which was demonstrated very well in the case reported. Orbital osteoma which is the most common benign tumour of the paranasal sinuses [14] may at time present a diagnostic challenge. This is occasioned at times by the nonspecific histological and radiological appearance which may result in poor characterization of the lesion.

Therapeutic indication depends on the course of tumour and the development of complications. This could range from mere observation with serial radiological follow-up to medical therapy with systemic corticosteroid and surgical intervention. The surgical option adopted in this case met with the basic tenets of operative intervention using the treatment protocol proposed by Chen and Noordhoff [15]. There was an obvious neurological effect as demonstrated by the progressive reduction in the visual acuity as well as the cosmetically unacceptable degree of proptosis; even more so for an unmarried young man. Although there was no visual improvement postoperatively despite postoperative administration of corticosteroid, this may not be surprising because of the large interval between the onset of symptoms and performance of surgical orbital decompression. However, part of the patient expectation was met as shown by the reduction in the degree of proptosis.

Complete resection of the lesion was not possible in this case because the entire posterior orbit was filled with the lesion. We concentrated on a curettage to provide enough room for repositioning of the globe, using a lateral orbitotomy approach which is associated with less morbidity and quick recovery. Cranio-orbital shaping is an acceptable mode of surgical treatment for fibrous dysplasia when it may not be possible to remove the pathological bone completely.

## Conclusion

Monostotoic fibrous dysplasia of the orbit causing neuro-ophthalmic complications associated with compressive mass effect should be considered in the differential diagnosis of slowly progressive proptosis in young adults.

## Competing interests

The authors declare that they have no competing interests.

## Authors' contributions

COB took part in the surgery and was a major contributor to preparing the manuscript. AOF, JKC and VNO took part in the surgery. OOK took part in the surgery and was a major contributor to preparing the manuscript. AOO performed the histological examination of the specimen. AIA and AMB were major contributors to the manuscript preparation. All authors read and approved the final manuscript.

## Consent

Written informed consent was obtained from the patient for publication of this case report and accompanying images. A copy of the written consent is available for review by the Editor-in-Chief of this journal.
